# Prevalence of Osteoporosis and Low Bone Mass in Older Chinese Population Based on Bone Mineral Density at Multiple Skeletal Sites

**DOI:** 10.1038/srep25206

**Published:** 2016-05-04

**Authors:** Yi-Chien Lu, Ying Chin Lin, Yen-Kuang Lin, Yi-Jui Liu, Kwang-Hwa Chang, Poon-Ung Chieng, Wing P. Chan

**Affiliations:** 1 Department of Radiology, School of Medicine, College of Medicine, Taipei Medical University, Taipei 110, Taiwan, Republic of China; 2 Department of Health Management Center, Wan Fang Hospital, Taipei Medical University, Taipei 116, Taiwan, Republic of China; 3 Department of Family Medicine, Shuang Ho Hospital, Taipei Medical University, New Taipei City 235, Taiwan, Republic of China; 4 Department of Family Medicine, School of Medicine, College of Medicine, Taipei Medical University, Taipei 110, Taiwan, Republic of China; 5 Biostatistics Center, Taipei Medical University, Taipei 110, Taiwan, Republic of China; 6 Graduate Institute of Nursing, Taipei Medical University, Taipei 110, Taiwan, Republic of China; 7 Department of Automatic Control Engineering, Feng Chia University, Taichung 407, Taiwan, Republic of China; 8 Department of Physical Medicine and Rehabilitation, Wan Fang Hospital, Taipei Medical University, Taipei 116, Taiwan, Republic of China; 9 Graduate Institute of Injury Prevention and Control, College of Public Health and Nutrition, Taipei Medical University, Taipei 110, Taiwan, Republic of China; 10 Department of Radiology, Taiwan Adventist Hospital, Taipei 105, Taiwan, Republic of China; 11 Department of Radiology, Wan Fang Hospital, Taipei Medical University, Taipei 116, Taiwan, Republic of China

## Abstract

Diagnosis of osteoporosis is based on bone mineral density (BMD) measurement, which is site dependent and commonly discordant between measurement sites. We aimed to determine the prevalence of osteoporosis diagnosed based on BMD T-scores measured by dual-energy x-ray absorptiometry (DXA) at different sites: the lumbar spine (LS) alone, femoral neck (FN) alone, or both. A total of 1712 women and 2028 men with LS and FN BMD measurements were enrolled. Over 50% discordance was found between osteoporosis classifications based on T-scores measured at the LS and FN. Use of the lowest T-scores measured at both the LS and right and left FN (rather than one site) significantly increased the prevalence of osteoporosis from 4.03 to 10.75% in postmenopausal women and 1.82 to 4.29% in men aged ≧50 years (*p* < 0.001). The trends of overall and age-adjusted prevalence of osteoporosis were similar in women and men. Osteoporosis was diagnosed at a higher rate if the USA reference rather than the Asia reference was used to calculate the T-score (26.64% vs. 10.75%). In conclusion, diagnosis based on the lowest T-score from multiple site BMD measurement can increase the prevalence of osteoporosis, demonstrating the higher sensitivity of the multiple site measurement strategy.

Fragility fracture caused by osteoporosis, predominantly in the hip and spine, is a serious health problem in the elderly population^1^,^2^. The burden of osteoporosis is reflected significantly by the ever increasing medical and hospital expenditures for fracture related problems worldwide^3^,^4^,^5^. A recent study reported that, for men and women, Taiwan has one of the highest hip fracture incidences (299/100,000 adjusted to the world population), higher than the United States (250/100,000)^6^. According to the Taiwan National Health Insurance Research Database, a total of 141,397 subjects aged ≧50 years had hip fractures diagnosed during the 2004 to 2011 period. Chen *et al.* estimated that this number of hip fractures will increase 2.7-fold over the next two decades (from 18,338 in 2010 to 50,421 in 2035) in Taiwan^7^.

Bone mineral density (BMD) decline is a major reason for the increased risk of fractures^8^. Many epidemiological studies have indicated that BMD values tend to decrease as people get older^9^,^10^,^11^. Dual-energy X-ray absorptiometry (DXA) is the standard and most precise technique for BMD measurement, and BMD is the standard measure for diagnosis of osteoporosis^12^. The World Health Organization (WHO) in 1994 first proposed diagnostic criteria for osteoporosis in postmenopausal white women^13^ and based the diagnosis of osteoporosis on T-score, which is the difference between the BMD value (in g/cm^2^) of an individual and the average BMD (expressed in standard deviation [SD] units) of a young adult in a reference population. A T-score of ≧−1; −2.5 < T-score <−1; T-score ≦−2.5, and T-score ≦−2.5 with one or more fragility fractures indicated normal bone mass, low bone mass, osteoporosis, and severe osteoporosis, respectively^14^,^15^.

The T-score is commonly discordant between the lumbar spine and hip^16^,^17^,^18^,^19^. Woodson classified T-score discordance into major and minor^16^. Major discordance means that the score indicates osteoporosis at one site but normal bone mass at the other site. Minor discordance means that the difference between the two sites is small, that is, the scores indicate osteoporosis at one site and low bone mass at the other site, or low bone mass at one site but not at the other^19^. Consequently, the International Society for Clinical Densitometry (ISCD) has recommended that BMD should be measured at both the lumbar spine and hip in all patients and that osteoporosis should be diagnosed on the basis of the lowest T-score^20^.

In Taiwan, the National Health Insurance reimburses the service fee for only one measurement. Therefore, most medical institutions conduct BMD measurements with DXA at only one site (mostly the lumbar spine). Because of the inconsistency of diagnosis between skeletal sites, measurement at only one site may underestimate the prevalence of osteoporosis. Some patients with fragility fractures at the femur or compression fractures in the lumbar spine have had BMDs indicating no osteoporosis. Moreover, reference population databases (mostly Asian vs. USA) are not used consistently among institutions performing DXA scans on scanners from the same company.

According to the WHO, the lifetime risk of low-impact fracture (at wrists, hips, or vertebrae) among postmenopausal women is 30–40% in developed countries^21^. Since the incidence of hip fracture is higher in Taiwan than the USA^6^, we followed the 2007 ISCD recommendation to routinely measure the BMD at the lumbar vertebrae and at both the right and left proximal femurs of each patient. In this study, we aimed to determine whether the prevalence of osteoporosis determined by using multiple site DXA measurements differs from that determined by a single site measurement. In addition, we compared the prevalence of osteoporosis diagnosed on the basis of T-scores calculated using Asia reference data versus that calculated using USA reference data.

## Results

**Figure 1:**
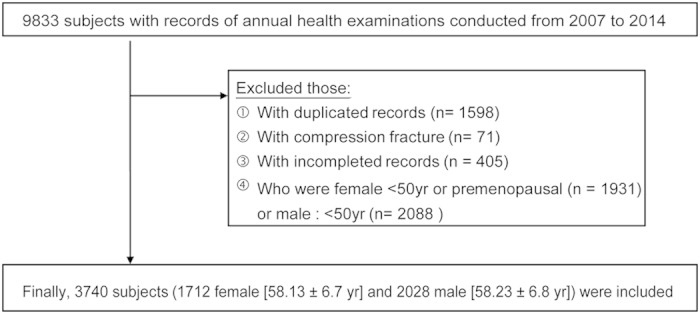
Flow chart of the study selection process.

A total of 3740 individuals (1712 postmenopausal women and 2028 men age ≧50 years) participated in the study (Fig. 1).

### Bone mineral density at multiple measurement sites

There was no significant diagnostic difference between T-score for the right and left femoral neck in both genders (Table 1). The T-score was significantly higher for the lumbar spine (−0.19 ± 1.47) than for the right and left femur (right femur, RF, −0.73 ± 1.04; left femur, LF, −0.73 ± 1.04) (*p* < 0.001). The overall average BMD was lower in women than in men by 12% at the lumbar spine (1.030 vs. 1.158) and by 10% at the right (0.809 vs. 0.891) and left (0.807 vs. 0.894) femoral neck. BMD values decreased gradually with age after the age of 50 years at each measurement site, except the lumbar spine in men (Table 1).

### Discordance in diagnosis of osteoporosis at different measurement sites

**Figure 2:**
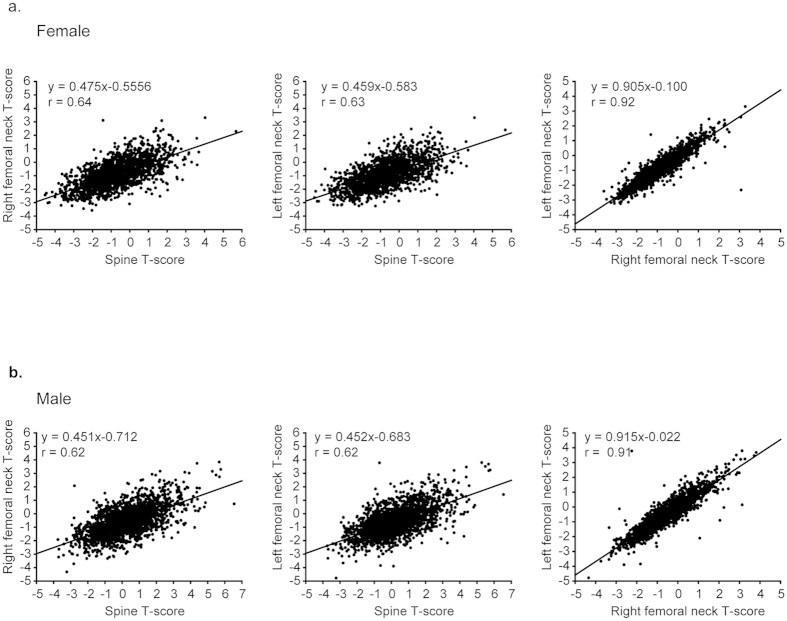
Correlation between lumbar spine, right femoral neck, and left femoral neck T-scores. Comparison of two different measurement-site T-scores in (**a**) 1712 female participants and (**b**) 2028 male participants.

The correlations between T-scores measured at the lumbar spine, and right and left femoral neck were, respectively, 0.64, 0.63, and 0.92 for women and 0.62, 0.62, and 0.91 for men (Fig. 2). T-scores from measurements at the lumbar spine and at the right and left femoral neck were moderately correlated in both sexes. In contrast, T-scores measured at the right and left femoral neck were highly correlated in both sexes. Diagnostic inconsistencies between measurement sites were noted (Table 2). Of 130 postmenopausal women age 50 years or older with a diagnosis of osteoporosis in the lumbar spine, 76% had no diagnosis of osteoporosis in either the right or left femoral neck. In addition, of 74 and 69 cases of osteoporosis diagnosed from measurements at the right and left femoral neck, over 50% were missed from measurements at the lumbar spine. Similarly, more than two-thirds of cases in men were misdiagnosed if based on the T-score from only a single site (lumbar spine vs. femur). These results suggest that diagnosis based on BMD measurement from only a single site underestimates the prevalence of osteoporosis.

### Combined multiple site measurements

**Figure 3:**
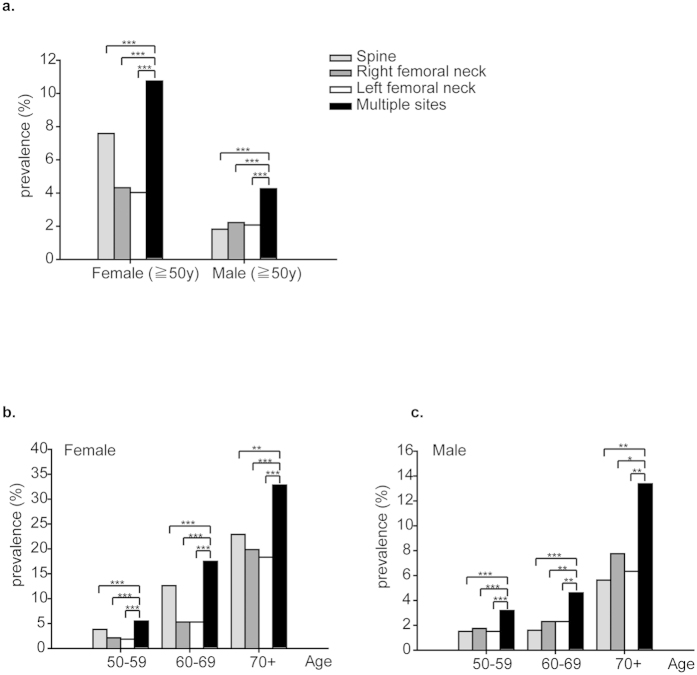
Comparison of the prevalence of osteoporosis as diagnosed on the basis of multiple site vs. single site measurements. (**a**) Prevalence of osteoporosis at different measurement sites for both women and men age 50 and older. (**b**) Prevalence of osteoporosis at different measurement sites for each age group of females and (**c**) males. McNemar’s test was used to test the significant differences in prevalence of osteoporosis diagnosed by multiple site vs. single site measurements. The Bonferroni adjustment was used as a post hoc analysis (**p < 0.01; ***p < 0.001).

The WHO recommends basing the diagnosis of osteoporosis on the T-score measured at the femoral neck^21^, while the ISCD recommends basing it on the lowest T-score from measurements at the spine, femoral neck, total hip, or trochanter^20^. Using the lowest T-score from multiple sites significantly increased the prevalence of osteoporosis in postmenopausal women from 4.03 to 10.75% and in men aged 50 years and older from 1.82 to 4.29% (Fig. 3a, Table 3). We further assessed prevalence at different skeletal sites by age (10-year intervals) and sex. The prevalence of osteoporosis was higher when diagnosed on the basis of the lowest of the lumbar spine and femoral neck T-score (Fig. 3b,c). For example, in women over age 70, the prevalence of osteoporosis increased approximately 10% and 13–15% when the lowest multiple site T-score was used than when the lumbar spine and femoral neck T-scores, respectively, were used. A similar result was observed in men. In men over age 70, the prevalence of osteoporosis based on multiple site measurement was approximately 6–8% higher than that based on single site measurement.

Comparing the young adult Asian reference (T_Asia_) with the young adult USA/Northern Europe reference (T_USA_) (which were respectively calculated using the average BMDs of two reference populations; Table 3) for T-score-based osteoporosis assessment, we found a higher prevalence of osteoporosis and low bone mass using T_USA_ than T_Asia_ in both sexes. The prevalence of osteoporosis using T_USA_ and T_Asia_ was, respectively, 26.64% and 10.75% in women and 21.20% and 4.29% in men.

## Discussion

We retrospectively reviewed the medical records of those classified as osteoporotic based on DXA scans over the last eight years and evaluated the discordance between diagnoses based on T-score at different BMD measurement sites. We found that the discordance between diagnoses of osteoporosis based on lumbar spine and femoral neck T-scores ranged from 52% (in men) to 81% (in women). Moreover, multiple site BMD measurement with DXA scans significantly increased detection of osteoporosis by 6.72% in women and 2.47% in men when compared with single site measurement.

In our study, the correlation between diagnoses based on lumbar spine and femoral neck measurements was moderate (Fig. 2). The prevalence of T-score discordance between lumbar spine and hip is a common observation in DXA^17^,^19^,^22^,^23^. Mounach *et al.* found only 54% concordance between T-scores calculated using lumbar spine and total hip BMDs from 3479 patients as well as a minor discordance in 42% and major discordance in 4% of the study population^19^. Another retrospective study in Indian postmenopausal women also revealed that nearly 34.47% had a minor discordance and 16.67% had a major T-score discordance between hip and spine^22^. Additionally, the risk factors affecting diagnostic discordance were recently identified as older age, menopause, obesity, belated or premature menopause, and multiple pregnancies^17^,^19^,^22^.

Woodson has proposed five different reasons for the occurrence of discordance including physiologic factors, pathophysiologic factors, anatomic factors, artifacts, and technical problems^16^. Hip dominance can account for physiologic discordance. It has been reported that weight-bearing can increase BMD in the hip and femur^24^. This might explain why obesity is considered a risk for major discordance. Pathophysiologic discordance, also called secondary discordance, is associated with degenerative diseases, such as vertebral osteophytosis, vertebral end plate and facet sclerosis, osteochondrosis, and aortic calcification^25^,^26^. Anatomic discordance is ascribable to differences in bone envelope composition. For example, the T-score of the postero-anterior lumbar spine and supine lateral lumbar spine in the same subject are quite different. Artifactual discordance occurs when dense materials (such as the metal from zippers, coins, clips, etc.) are within the region of interest. Technical discordance is due to device errors, technician variability, or patients’ movements. In our study, this bias was probably negligible because our technicians certified by ISCD scrupulously followed the scan positioning and analysis guidelines of the ISCD^27^.

Diagnosis of osteoporosis is based on the lowest T-score at two or more sites of BMD measurement with DXA scans. Although the WHO and the European Society for Clinical and Economic Aspects of Osteoporosis and Osteoarthritis have stressed reliance on the femoral neck BMD for osteoporosis assessment^21^,^28^, other organisations including the National Osteoporosis Foundation (NOF) and the ISCD recommend BMD values from the lumbar spine, total hip, and femoral neck^27^,^29^. Blake *et al.* used a mathematical model to determine whether combining two BMD measurements (lumbar spine and femoral neck) improves fracture discrimination, and they showed little benefit to using a combination approach^30^. These results were further confirmed by a prospective meta-analysis reported by Kanis *et al.* in 2006^31^. However, a recent investigation reported the feasibility of using a combination of femur neck and lumbar spine BMD measures to assess hybrid 10-year absolute fracture risk^32^. Another cohort study in 16,505 Canadian women indicated that adding lumbar spine BMD measurement to a fracture prediction model including femoral neck measurement increases fracture prediction for the overall population. Age-adjusted results showed increased fracture prediction only in women aged 50–64 years but not in older women^33^. The National Health and Nutrition Examination Survey (NHANES), conducted by the National Center for Health Statistics, collected both lumbar spine and proximal femur BMD values to evaluate the health and nutrient status of the US population from 2005. The latest report using lumbar spine and femoral neck BMD data, measured by the Hologic QDR 4500A fan-beam DXA and collected in the NHANES 2005–2010 database, estimated that 15.4% of adult women and 4.3% of adult men 50 years and older in the US had osteoporosis^34^. As mentioned in their report, use of the non-institutionalized US population could have led to an underestimate of the true prevalence of osteoporosis in the population. Moreover, the use of different DXA manufacturers and reference databases could also have influenced the diagnosis of osteoporosis when compared with other studies. In our study, using the lowest T-score, the prevalence of osteoporosis increased from 4.03 to 10.75% in postmenopausal women and from 1.82 to 4.29% in men aged 50 years and older. Furthermore, the prevalence of osteoporosis was significantly increased in each age-stratified subgroup (Fig. 3).

The performance characteristics of BMD to predict fractures are at least as good as the performance characteristics of blood pressure to predict stroke^35^. It is well known that low bone mass is a major cause of fragility fracture. A nationwide epidemiological study conducted by the National Health Insurance Research Database in Taiwan showed that hip fracture incidence increased significantly by 30% from 1996 to 2002^36^. Chan *et al.* also reported that the total number of hip fractures increased by 25% from 1999 to 2010 in Taiwan^5^. Moreover, a worldwide systematic study has indicated that the hip fracture rate is higher in Taiwan than the US^6^. However, most medical institutions measure BMD with DXA at only one skeletal site, which underestimates the prevalence of osteoporosis and low bone mass as shown in our study.

The standard diagnostic classification for osteoporosis is based on the T-score (according to WHO criteria), which is defined as the number of standard deviations from the young adult population-based reference value for peak BMD^14^. The WHO and the NOF recommended using the femur reference value for American Caucasian women from NHANE III, if local reference data are unavailable. Nevertheless, recent studies have reported quite different peak BMD values among ethnicities^37^,^38^,^39^. For example, the peak lumbar spine and femoral neck BMD values for Chinese women are significantly lower than those for Caucasian women^37^. Moreover, Melamed *et al.* reported that switching from the standard reference value for the US white population to one for the South Indian population led to reclassification, with BMD measured at the total hip in 19% of participants and the lumbar spine in 40% of participants^40^. In our study, using the T_USA_ rather than the Asian based population-derived reference value (T_Asia_) increased the proportion of women and men with a diagnosis of osteoporosis and low bone mass (Table 3). In our study, osteoporosis was diagnosed on the basis of T-score (T_Asia_) in 10.75% of postmenopausal women and 4.29% of men aged 50 years and over, and osteoporosis diagnosed by T-score (T_USA_) in 26.64% of postmenopausal women and 21.20% of men aged 50 years and over. The prevalence of osteoporosis diagnosed using T_USA_ was closer to 30%, which is the prevalence in Caucasian postmenopausal women as estimated from measurements made at the spine, hip, or forearm according to WHO criteria^41^. When comparing the prevalence of low bone mass diagnosed by T_USA_ vs. T_Asia_, we found a similar prevalence (53.27% vs. 50.76%) in postmenopausal women and higher prevalence (57.94% vs. 41.72%) in men aged 50 years and older. These findings indicate that using different BMD reference values could influence the diagnosis and thereby affect subsequent clinical treatment. Hence, it is important to choose an appropriate reference value to calculate the T-score for osteoporosis diagnosis in our population.

Owing to the rising awareness of osteoporosis prevention and before local criteria are established in Taiwan, our results showed that T-scores based on multiple site BMD measurement and calculated with T_USA_ as reference increase the level of osteoporosis detection and prevalence, in accordance with the very high incidence of hip fracture in Taiwan and the 30–40% lifetime risk of low-impact fractures among postmenopausal women according to the WHO.

This study had several limitations. First, the study population was from a single institution. However, our institute is one of the largest institutes conducting BMD measurement at multiple sites in Taiwan. It was hard to enroll subjects from multiple centers as most institutions perform only single site BMD measurement. Second, all BMD measurements were performed on a single DXA scanner. The Lunar Asia reference database contains data from a variety of countries, including China, Japan, and Korea. Significant differences in BMD between genders, ethnicity, and geographic regions have been reported^37^,^42^,^43^,^44^. A previous report showed lower BMD values in Chinese than in Japanese and Koreans^10^, which would lead to miscalculation of the true prevalence of osteoporosis in our country.

In summary, our results, for the first time, provide evidence that diagnosis based on the lowest T-score from multiple site BMD measurement and calculation with the Caucasian reference according to WHO criteria can increase the level of osteoporosis detection and prevalence in a Chinese population aged 50 years or older, which is close to the 30% in Caucasian postmenopausal women.

## Materials and Methods

### Study design and participants

We retrospectively reviewed the medical records of 9833 patients who received annual health examinations with DXA scans at our institute between January 1, 2007 to December 31, 2014, and fulfilled the following criteria: (1) age 50 years and older, (2) DXA by a single scan to determine BMD at multiple sites (lumbar spine and both right and left femoral neck), and (3) postmenopausal if female, (4) no history of compression fracture, (5) Chinese/Taiwanese nationality. Only the first medical record of patients with multiple records was used. All the participants were grouped by sex and age at intervals of 10 years (50–59, 60–69, and 70+). Of these, 6093 patients were excluded: 1598 follow-up examinations, 71 patients with compression fracture at the lumbar spine, 405 patients with incomplete records (e.g., missing BMD data and women with no record of menstrual cycle status), and 4019 patients under age 50 or premenopausal. Finally, a total of 3740 patients participated in this study.

The protocol of this study was approved by the Taipei Medical University-Joint Institutional Review Board (TMU-JIRB) after an expedited review process. Informed consent was waived due to the retrospective nature of this study.

### Bone mineral density measurements

Multiple site BMD examination (lumbar spine and right and left femoral neck) was routinely performed, beginning in 2007 at our institution, on a DXA densitometer (Lunar Prodigy, version 9.1; GE Healthcare, Madison, WI) by three experienced technicians certified by the ISCD. One senior technician performed majority of cases. Daily calibration was performed with a GE-provided phantom (calibration standard) and the maximum coefficient of variation (CV%) of BMD measurement was 0.34%. The precision of BMD measurement for lumbar spine and femoral neck of 30 patients twice, scanned with repositioning within the same day, was 0.61% and 1.07%, respectively. And the least significant change of bone mineral content for lumbar spine and femoral neck was 1.68% (root mean square standard deviation (RMS-SD), 0.017 g/cm^2^) and 2.97% (0.025 g/cm^2^), respectively. Bone density was measured at the lumbar spine (L_I_-L_4_) and both the right and left proximal femurs on a postero-anterior scan. Each patient was examined in the supine position with an accessory for measuring the BMD value of lumbar spine. Both knees were bent slightly inward and legs were placed flat with each foot strapped to a foot holder for measuring the BMD value of the femur. Each patient was placed in the middle and parallel to the long axis of the examination table. The BMD values are expressed in g/cm^2^. All measurements were performed on a single scanner.

To clarify whether the diagnoses of osteoporosis by assessment at different measurement sites were concordant, we used the WHO classification system, which defined osteoporosis on the basis of measurements at the spine, and the right and left femoral neck. According to the WHO criteria, we diagnosed *osteoporosis* (if T-score ≦−2.5), *low bone mass* (if −2.5 <T-score <−1), or *normal* BMD (if T-score ≧−1). The T-score was calculated using a standard formula: T-score = (BMD – reference value [peak BMD in a young normal population])/one standard deviation. The reference values for Asia and USA/Northern Europe were provided by GE Healthcare (Madison, WI). We calculated the T-score for each subject using the two different reference values. One senior radiologist certified by the ISCD, and following ISCD standards, interpreted the BMD data in 95% of the DXA reports.

### Statistical analysis

The database was established by Excel software. A paired *t* test was used for comparing the T-score between the lumbar spine and right and left femoral neck. The relationship between the lumbar spine T-score and femoral neck T-score was analyzed by linear regression. McNemar chi-square test was used for testing the significance of differences in prevalence of osteoporosis between multiple site and single site measurements. Bonferroni adjustment was adopted as a post hoc procedure to account for the multiple testing issues. Statistical analyses were performed using SAS v.9.3 (SAS Institute Inc, Cary, NC) and a value of *p* < 0.05 was considered statistically significant.

## Additional Information

**How to cite this article**: Lu, Y.-C. *et al.* Prevalence of Osteoporosis and Low Bone Mass in Older Chinese Population Based on Bone Mineral Density at Multiple Skeletal Sites. *Sci. Rep.* **6**, 25206; doi: 10.1038/srep25206 (2016).

## Figures and Tables

**Table 1:** Multiple site BMD and T-score* (mean ± SD) measurements in the current study population.

Age group	Number	Lumbar spine		Right femoral neck		Left femoral neck	
		BMD (g/cm^2^)	T-score	BMD (g/cm^2^)	T-score	BMD (g/cm^2^)	T-score
**Total population**	**3740**	**1.099 ± 0.180**	**−0.19 ± 1.47**	**0.854 ± 0.130**	**−0.73 ± 1.04#**	**0.854 ± 0.130**	**−0.73 ± 1.04#**
50–59	2451	1.113 ± 0.169	−0.08 ± 1.39	0.870 ± 0.126	−0.60 ± 1.02 #	0.872 ± 0.127	−0.58 ± 1.02 #
60–69	1016	1.082 ± 0.192	−0.34 ± 1.57	0.834 ± 0.127	−0.90 ± 0.99 #	0.832 ± 0.126	−0.92 ± 0.97 #
70+	273	1.046 ± 0.213	−0.64 ± 1.73	0.778 ± 0.137	−1.35 ± 1.07 #	0.779 ± 0.142	−1.34 ± 1.11 #
**Females**	**1712**	**1.030 ± 0.163**	**−0.69 ± 1.36**	**0.809 ± 0.121**	**−0.88 ± 1.00 #**	**0.807 ± 0.119**	**−0.90 ± 0.99 #**
50–59	1129	1.063 ± 0.156	−0.41 ± 1.30	0.833 ± 0.118	−0.68 ± 0.98 #	0.833 ± 0.116	−0.68 ± 0.97 #
60–69	452	0.977 ± 0.156	−1.13 ± 1.30	0.775 ± 0.108	−1.17 ± 0.90	0.769 ± 0.103	−1.22 ± 0.86
70+	131	0.927 ± 0.158	−1.54 ± 1.31	0.718 ± 0.116	−1.64 ± 0.97	0.716 ± 0.114	−1.65 ± 0.95
**Males**	**2028**	**1.158 ± 0.172**	**0.23 ± 1.44**	**0.891 ± 0.125**	**−0.61 ± 1.05 #**	**0.894 ± 0.126**	**−0.58 ± 1.05 #**
50–59	1322	1.155 ± 0.168	0.20 ± 1.40	0.901 ± 0.125	−0.52 ± 1.04 #	0.905 ± 0.126	−0.49 ± 1.05 #
60–69	564	1.165 ± 0.175	0.29 ± 1.47	0.885 ± 0.119	−0.68 ± 1.00 #	0.886 ± 0.119	−0.68 ± 0.99 #
70+	142	1.155 ± 0.198	0.20 ± 1.65	0.834 ± 0.132	−1.09 ± 1.10 #	0.837 ± 0.140	−1.06 ± 1.17 #

^*^T-score was calculated using an Asian-based young adult population-derived reference value provided by GE Healthcare, Madison, WI.

^#^Statistically significant difference (*p* < 0.05) *vs.* T-score at the lumbar spine using a paired *t-test.*

**Table 2:** Frequency of discordant diagnosis based on T-score* measured at various sites according to WHO criteria.

Positive for osteoporosis		Negative for osteoporosis		
Site	Number	Spine# (%)	Right femur# (%)	Left femur# (%)
**Female**				
Spine	130	–	100 (76.92)	97 (74.62)
Right femur	74	44 (59.46)	–	28 (37.84)
Left femur	69	36 (52.17)	23 (33.33)	–
**Male**				
Spine	37	–	28 (75.68)	25 (67.57)
Right femur	47	38 (80.85)	–	22 (46.81)
Left femur	42	30 (71.43)	17 (40.48)	–

^*^T-score was calculated using the average BMD of a young adult reference population in Asia.

^#^Results (expressed as percentage) are in parenthesis. For example, 130 female participants were diagnosed as having osteoporosis based on the T-score at the lumbar spine. Among these patients, however, 100 women (76.92%) and 97 women (74.62%) were not diagnosed as having osteoporosis based on measurement at the right and left femoral neck, respectively.

**Table 3:** Comparison of osteoporosis prevalence diagnosed by T-scores at different sites, each calculated using two reference populations.

Diagnosis	Spine		Right femur		Left femur		Multiple sites	
	T_Asia_*	T_USA_#	T_Asia_	T_USA_	T_Asia_	T_USA_	T_Asia_	T_USA_
**Females (n = 1712)**								
*Osteoporosis (age* ≧*50 y)*	**7.59**	**18.40**	**4.32**	**13.39**	**4.03**	**14.50**	**10.75**	**26.64**
50–59	3.81	12.05	2.13	8.15	1.86	8.24	5.49	18.07
60–69	12.61	28.32	5.31	17.70	5.31	20.80	17.48	38.27
70 +	22.90	38.93	19.85	43.51	18.32	46.56	32.82	60.31
*Low bone mass (age* ≧*50 y)*	**35.63**	**41.65**	**43.28**	**54.62**	**44.22**	**54.74**	**50.76**	**53.27**
50–59	31.62	39.77	37.29	52.79	37.64	53.32	47.65	55.62
60–69	43.58	46.24	54.87	62.17	56.19	62.39	58.41	52.88
70+	42.75	41.98	54.96	43.51	59.54	39.69	51.15	34.35
*Normal (age* ≧*50 y)*	**56.78**	**39.95**	**52.28**	**31.99**	**51.64**	**30.76**	**38.49**	**20.09**
50–59	64.57	48.18	60.58	39.06	60.41	38.35	46.86	26.31
60–69	43.81	25.44	39.38	19.69	38.27	16.59	24.12	8.85
70+	34.35	19.08	25.19	12.98	22.14	13.74	16.03	5.34
**Males (n = 2028)**								
*Osteoporosis (age* ≧*50 y)*	**1.82**	**6.41**	**2.22**	**16.07**	**2.07**	**15.15**	**4.29**	**21.20**
50–59	1.51	5.52	1.74	14.15	1.51	12.78	3.18	18.76
60–69	1.60	6.91	2.30	15.25	2.30	16.31	4.61	21.28
70+	5.63	12.68	7.75	36.62	6.34	32.39	13.38	43.66
*Low bone mass (age* ≧*50 y)*	**17.90**	**33.88**	**34.70**	**54.57**	**33.93**	**54.74**	**41.72**	**57.94**
50–59	18.46	35.48	32.07	54.08	31.16	54.54	39.86	59.23
60–69	16.67	31.03	37.06	58.51	36.17	56.56	44.50	59.40
70+	17.61	30.28	47.18	41.55	50.70	48.59	47.89	40.14
*Normal (age* ≧*50 y)*	**80.28**	**59.71**	**63.07**	**29.36**	**63.91**	**30.11**	**53.99**	**20.86**
50–59	80.03	59.00	66.11	31.69	67.25	32.60	56.96	22.01
60–69	81.74	62.06	60.11	25.71	61.35	26.95	50.89	19.33
70+	76.76	57.04	44.37	21.13	42.96	19.01	38.73	16.20

^*^T-score was calculated using a young adult Asian population reference value provided by GE Healthcare, Madison, WI.

^#^T-score was calculated using a young adult USA/Northern Europe population reference value provided by GE Healthcare, Madison, WI.
